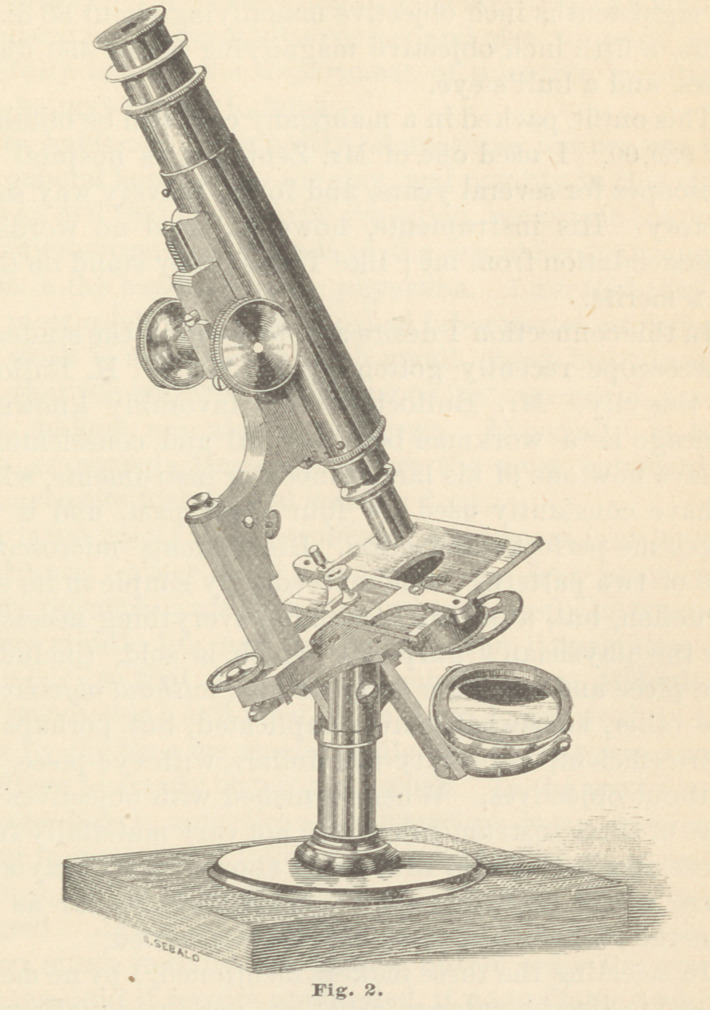# The Microscope in Daily Practice, (Second Paper)

**Published:** 1875-10

**Authors:** I. N. Danforth

**Affiliations:** Lecturer on Pathology in Rush Medical College, Chicago


### THE MICROSCOPE IN DAILY PRACTICE.

(SECOND PAPER.)

By I. N. DANFORTH, M.D.,

Lecturer on Pathology in Rush Medical College, Chicago.

It seems to be generally believed by physicians and students that a good microscope must necessarily be a very costly affair ; hence the majority of those who ought to possess microscopes are "waiting" till they are rich enough to afford it. Now a microscope is one of the instruments that the student should purchase first ; it should be the constant companion of his student-days, so that when he commences practice he shall be as familiar with medical microscopy as he is with any other means of diagnosis or scientific research. It is a poor time to study practical microscopy when the physician finds himself involved in a busy practice, but most of us can find time enough in our earlier years to at least make ourselves quite successful *amateurs*.

Until within a few years past a good microscope at a reasonable cost was a thing almost unknown. During the last decade, however, several of our best makers have, with the aid of practical microscopists, placed within our reach instruments of excellent quality and comparatively low price.

Through the courtesy of Mr. J. G. Langguth, the well-known optician of this city, I am able to present illustrations of two very excellent physicians' microscopes, either of which will furnish every requisite for ordinary diagnostic purposes, or pathological research.

Fig. 1 represents the students' microscope, manufactured by Mr. R. B. Tolles, of Boston. This is one of the best low-priced instruments ever offered. Some years ago, Mr. Tolles conceived the idea of furnishing a good instrument at a low price, and in order to successfully carry out his scheme, he sought the advice of several practical microscopists of Boston and vicinity, notably

Fig. 1.

Professors Oliver Wendell Holmes and the late Jeffries Wyman. The result of their deliberations and experiments was the beautiful instrument of which the accompanying wood-cut is a very correct illustration. The base, upright and curved arm, are of iron, handsomely japanned; the body of the instrument is nickel-plated, and is fixed to the curved arm by a trunnion joint, by means of which it can be placed in any position from vertical to horizontal; it is furnished with a B eye-piece, an inch, and a quarter inch objective, the former giving about 80 and the latter about 350 diameters; plain (that is, fixed) stage, revolving diaphragm, and concave and plane mirrors. For the illumination of opaque objects, the mirror is removed to an upright stand. Coarse adjustment is effected by a rack and pinion movement, fine adjustment by a very fine screw and movable plate on the stage, which is sufficiently delicate for very high powers.

The price of this instrument, complete, together with a walnut case, is seventy dollars. To say that it is made by Mr. Tolles is a sufficient guarantee for its excellence.

Fig. 2 represents the new students' microscope, designed and manufactured by Mr. Joseph Zentmeyer, of Philadelphia, a gentleman long and favorably known to microscopists all over the world. This instrument bears a striking resemblance to many of those manufactured in Europe. It is mounted on a polished mahogany base, made to exactly fit the case, thus rendering all further packing unnecessary. It is capable of assuming any position from vertical to horizontal; the body of the instrument is rather short, but is capable of elongation by means of a draw tube. The stage is of glass, and is freely movable in all directions; below the stage is an

Fig. 2.

attachment for accessories, to which is fixed a revolving diaphragm, and below this attachment, two mirrors, plane and concave, so arranged as to allow free lateral movement—coarse adjustment by rack and pinion, which moves the body alone; fine adjustment by a fine micrometer screw acting on a lever which moves both the supporting arm and the body. The perforation in the bar is for the stem of the illuminating lens or "bull's-eye." Accompanying this instrument is a B eye-piece, an eight-tenths inch objective magnifying about 80 diameters, a fifth inch objective magnifying about 450 diameters, and a bull's-eye.

This outfit, packed in a mahogany case, can be obtained for $85.00. I used one of Mr. Zentmeyer's hospital microscopes for several years, and found it every way satisfactory. His instruments, however, need no words of commendation from me; like Tolles', they stand on their own merits.

In this connection I desire also to mention the students' microscope recently gotten up by Mr. W. H. Bulloch, of this city. Mr. Bulloch is very favorably known in Chicago as a workman both skillful and conscientious. I have now one of his large binocular instruments, which I have constantly used for four years past, and it has given me perfect satisfaction. His students' microscopes are of two patterns: one, exceedingly simple in its construction, but, after all, combining everything necessary for the physician's purposes, which is sold, (including eye-piece and case), for forty dollars, *without objectives;* the other, somewhat more complicated, but perhaps no more efficient, costs sixty-five dollars, with eye-piece, but without objectives. When furnished with objectives the cost of these instruments would not vary materially from those alluded to, and illustrated above; and I may add, there would be little choice between them so far as the general wants of the physician are concerned.

In selecting the three makers mentioned, I by no means intend to discriminate against others, perhaps equally good in every respect; but in the first place, in order to render this paper of any practical value to the readers of the JOURNAL AND EXAMINER in the selection of microscopes, it is necessary to recommend the productions of *somebody;* and in the next place, it is but right and natural that I should select the instruments of those makers with whom I am best acquainted.

With either of the three instruments above mentioned, the practicing physician ought to be able to do all things requisite for diagnostic purposes, and also to work successfully in whatever department of scientific investigation he may choose to labor.

The habit of buying costly microscopes has become far too general here in this country, and notably in this city. It is an evil that ought to be remedied, because it confines the microscope to the hands of the few, whereas it ought to be in the hands of every physician. I am told that in the most celebrated physiological laboratories of Europe the work is mainly done with small, cheap instruments, and that the majestic and gorgeous instruments of American makers are almost unknown. Especially is this true as regards Germany, where the most painstaking and accurate histological work is done.

A large, costly and complicated microscope is an actual hindrance, rather than a help. In the first place, not many physicians are able to purchase a costly instrument before middle life, and by that time the "fingers are all thumbs," so that delicate manipulations are difficult if not impossible; and most of us begin to get weak in the eyes by the time we reach middle life, so that we cannot sit down to steady histological work. In the next place, a large, heavy, complicated microscope is an unwieldy thing to manage; it takes too much time to get it ready for use, and is, therefore, rather more than likely to go unused. Again, so complicated and delicate a machine is too much in danger of getting out of order for every-day use, and if repairs are needed, it necessitates packing the instrument, and sending it away, sometimes for a long distance, which means loss of time, provoking delays, and outlay of money.

Lastly, unless one is pretty constantly in the practice of microscopy, the results obtained with the more elaborate instruments are far less satisfactory than with the simpler forms which are more easily managed. The ideal physicians' microscope is one that is not discouragingly costly; one that can be easily handled, rapidly and accurately adjusted, and one that can be quickly put in its place when the work in hand is done. Either of the instruments described in this paper come very near the realization of that ideal, combining as they do cheapness, simplicity and the greatest utility.

(To be continued.)

## Figures and Tables

**Fig. 1. f1:**
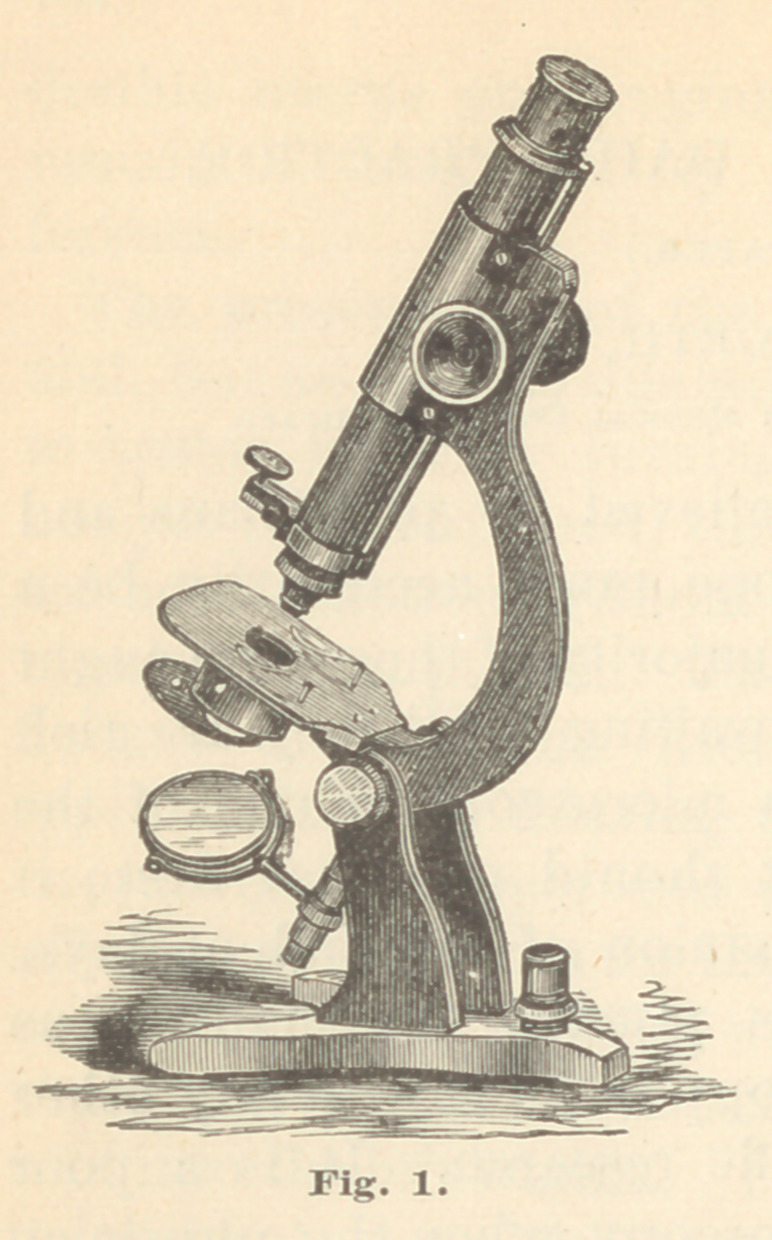


**Fig. 2. f2:**